# Synthesis of Lactic Acid-Based Thermosetting Resins and Their Ageing and Biodegradability

**DOI:** 10.3390/polym12122849

**Published:** 2020-11-29

**Authors:** Lara Lopes Gomes Hastenreiter, Sunil Kumar Ramamoorthy, Rajiv K. Srivastava, Anilkumar Yadav, Akram Zamani, Dan Åkesson

**Affiliations:** 1 Swedish Centre for Resource Recovery, Academy for Textile, Engineering and Business, University of Borås, 501 90 Borås, Sweden; laralghastenreiter@gmail.com (L.L.G.H.); sunil.kumar@hb.se (S.K.R.); akram.zamani@hb.se (A.Z.); 2 Department of Textile Technology, Indian Institute of Technology Delhi, New Delhi 110 016, India; rajiv@textile.iitd.ac.in (R.K.S.); anil20yadav@gmail.com (A.Y.)

**Keywords:** bio-based polymer, thermoset resins, lactic acid, ageing, biodegradation

## Abstract

The present work is focused on the synthesis of bio-based thermoset polymers and their thermo–oxidative ageing and biodegradability. Toward this aim, bio-based thermoset resins with different chemical architectures were synthesized from lactic acid by direct condensation with ethylene glycol, glycerol and pentaerythritol. The resulting branched molecules with chain lengths (n) of three were then end-functionalized with methacrylic anhydride. The chemical structures of the synthesized lactic acid derivatives were confirmed by proton nuclear magnetic resonance spectroscopy (^1^H-NMR) and Fourier transform infrared spectroscopy (FT–IR) before curing. To evaluate the effects of structure on their properties, the samples were investigated by differential scanning calorimetry (DSC), thermogravimetric analysis (TGA) and the tensile testing. The samples went through thermo-oxidative ageing and biodegradation; and their effects were investigated. FT-IR and ^1^H-NMR results showed that three different bio-based resins were synthesized using polycondensation and end-functionalization. Lactic acid derivatives showed great potential to be used as matrixes in polymer composites. The glass transition temperature of the cured resins ranged between 44 and 52 °C. Pentaerythritol/lactic acid cured resin had the highest tensile modulus and it was the most thermally stable among all three resins. Degradative processes during ageing of the samples lead to the changes in chemical structures and the variations in Young’s modulus. Microscopic images showed the macro-scale surface degradation on a soil burial test.

## 1. Introduction

In recent years, bio-based polymers, prepared from renewable resources, have been of much interest to researchers from both the academic sector and industries, in order to find a substitute for petroleum-based polymers. The extensive use of fossil resources and the dependence on them is becoming an issue in the polymer industry owing to the challenges brought about by the fast depletion of the fossil resources, the frequent price fluctuations and the increasing demand for sustainable materials. Fossil resources-based polymers raise several environmental concerns, namely, those of sustainability, carbon dioxide emissions, disposal and recyclability. In an attempt to decrease the dependency on fossil resources, polymer researchers have been rapidly developing bio-based thermoplastics. In comparison with the rapid developments in bio-based thermoplastics, considerably less research has been done on bio-based thermosets. However, researchers have been developing bio-based thermoset resins from monomers or raw materials, such as soybean oils, linseed oil and lactic acid (2-hydroxypropanoic acid) [1,2,3].

Lactic acid can be produced by fermentation of sugar-based carbohydrates or by chemical synthesis [4]. Studies have been published on synthesis of polymers from lactic acid or lactide and branching molecules. The demand for lactic acid has grown because of its use in the synthesis of polylactic acid (PLA), a biodegradable and biocompatible polymer, well established in the bio-based polymer market. This polymer has been employed in many industrial applications, such as packaging—to reduce the risk of pathogen contamination in fruit and vegetable packages—and biomedical materials [5,6].

However, for many structural applications, thermosetting resins are necessary. Most commercial thermosetting resins on the market today are produced from non-renewable materials and the development of bio-based thermoset resins is on-going [7]. A number of studies show that the thermoset resins can be synthesized from lactic acid [1,8,9,10,11,12,13]. These resins can potentially be used for composite applications [14,15,16,17] or for coating applications [18]. Several attempts have involved a two-step synthesis where lactic acid is reacted with a core molecule in a direct condensation (poly-condensation) followed by an end-functionalization of the branches where reactive double bonds are introduced. Addition of the functional molecule to the branches changes the physiochemical properties of the polymer. The resins can subsequently be crosslinked by free radical reactions. Jahandideh and Muthukumarappan reviewed star-shaped thermoset lacitic acid-based resins [19]. This study is a continuation of our previous work and the aim of this study was to investigate the effects of chemical structure on the properties of three polyester resins synthesized from lactic acid with different core-molecules—ethylene glycol, glycerol and pentaerythritol. The cured resins were characterized using differential scanning calorimetry (DSC), thermogravimetric analysis (TGA), Fourier transform infrared spectroscopy (FT-IR) and the tensile test.

It is well known that polyesters are prone to hydrolysis which may determine their long-term properties and which applications they can be used for [6,7]. The degradation rate of the polymer can be altered by changing the ratio of hydrophilic groups to the hydrophobic groups. This gives significant control over the degradation rate and increases the control over the release of a certain component [19]. A useful way to ascertain the material’s long-term applications is by analyzing its responses through ageing and biodegradation tests. During a plastic product’s service life, it can be exposed to various factors, such as temperature, UV radiation, oxygen, ozone moisture, fuels, oils and chemicals. Those things will affect their mechanical properties, such as stiffness and strength, and limit their operating times. Several research studies have consequently been performed to understand the effects of ageing and biodegradation on thermoplastic resins [20,21,22]. PLA ageing and stabilization through an antioxidant was studied while performing accelerated thermo-oxidative ageing of PLA over its glass transition temperature in order to simulate the polymer’s service life [20,21]. Biodegradation of PLA was studied in detail earlier through a soil burial test [22].

Three bio-based thermosets were synthesized with different core molecules in order to examine the effects of chemical architecture on the biodegradation and hydrolytic stability. The resins were exposed to biodegradation and ageing tests and their responses were characterized by infrared spectroscopy and mechanical tests.

## 2. Materials and Methods

### 2.1. Materials

L-Lactic acid (88–92%, Sigma-Aldrich) was purified by using a rotary evaporator. Ethylene glycol (99.5%; Sigma-Aldrich, St. Louis, MO, USA), glycerol (99%; Acros Organics, Waltham, MA, USA) and pentaerythritol (98%; Sigma Aldrich) were used as received. Toluene was used as a solvent (99.99%; Fisher Scientific, Waltham, MA, USA) and methanesulfonic acid (98%; Alfa Aesar, Haverhill, MA, USA) was used as the catalyst in the condensation reaction. For end-functionalization of the intermediate products, methacrylic anhydride (94%; Alfa Aesar) was used as a reagent. Hydroquinone (99%; Across Organics, supplied by Fisher Scientific) was used as an inhibitor during the end-functionalization reaction.

### 2.2. Synthesis

**Figure 1:**
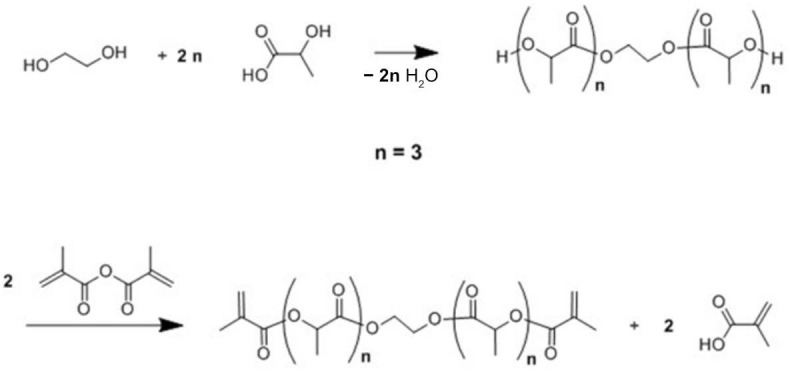
Reaction scheme of the first and the second stages for the synthesis of methacrylate functionalized ethylene glycol/lactic acid resin.

**Figure 2:**
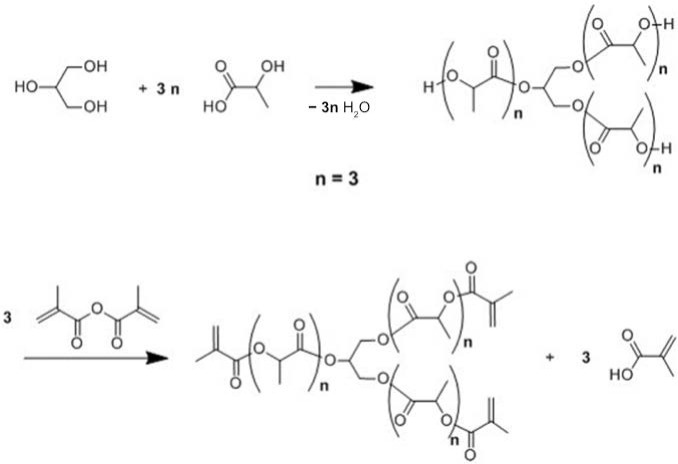
Reaction scheme of the first and the second stages for the synthesis of methacrylate functionalized glycerol/lactic acid resin.

**Figure 3:**
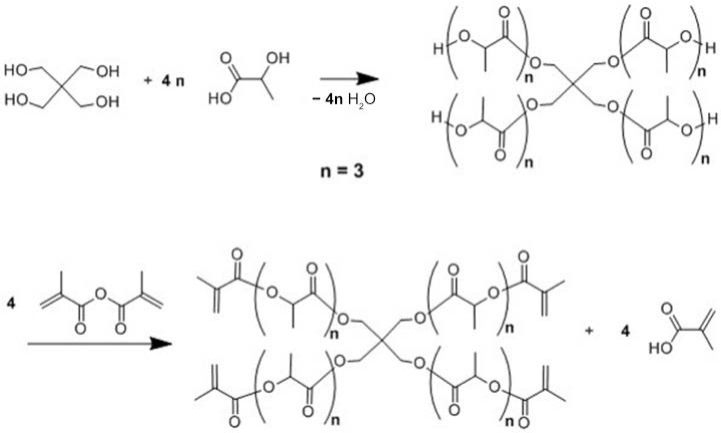
Reaction scheme of the first and the second stages for the synthesis of methacrylate functionalized pentaerythritol/lactic acid resins.

Three different bio-based thermoset resins were synthesized using lactic acid. The water from the lactic acid was first evaporated using a rotary evaporator and then stored dry at room temperature. Following this, the synthesis was then performed in two stages. In the first stage, namely, the condensation reaction stage, linear oligomers were prepared by reacting the lactic acid with ethylene glycol (EG/LA), and star-shaped oligomers were obtained by reacting lactic acid with glycerol (GLY/LA) and with pentaerythritol (PENTA/LA). The reaction schemes of the condensation reactions between lactic acid (LA) and ethylene glycol (EG), LA and glycerol (GLY) and LA and pentaerythritol (PENTA) are shown in Figure 1, Figure 2 and Figure 3. A chain length of 3 LA monomers was maintained for the oligomers. Thus 6, 9 and 12 mol of lactic acid were added for each mole of EG and GLY and PENTA.

In the second stage (the end-functionalization stage), the resin was reacted further with methacrylic anhydride. The result was three distinct resins with different chemical architectures.

#### 2.2.1. Stage 1: Direct Condensation Reaction

In total, 0.24 mol of ethylene glycol was added to 1.44 mol of lactic acid diluted in 50 g of toluene containing 0.1 wt % of methanesulfonic acid, which was used as catalyst. All components were placed in a three-neck round bottom flask equipped with a magnetic stirrer, a nitrogen inlet and an azeotropic distillation apparatus. The solution was heated for 2 h in an oil bath with a set temperature of 145 °C. The water produced in the reaction was collected by azeotropic distillation. After the initial reaction, the temperature was raised to 165 °C for further 2 h, and finally increased to 195 °C for 1 h under constant stirring.

The same procedure was followed to prepare other oligomers by reacting 0.16 mol of glycerol with 1.44 mol of lactic acid and 0.12 mol of pentaerythritol with 1.44 mol of lactic acid.

#### 2.2.2. Stage 2: End-Functionalization of the Oligomers

The oligomers were end-functionalized with 0.5 mol of methacrylic anhydride. Methacrylic anhydride was added slowly in drops using a dropping funnel. This introduces reactive double bonds in the structure, which enables the resin to be crosslinked. Hydroquinone (0.1 wt %) was also added, in order to stabilize the reaction and avoid premature curing. The mixture was stirred continuously for 4 h in an atmosphere of nitrogen with constant temperature maintained at 90 °C.

The by-product methacrylic acid that had formed after the second stage and the toluene were removed by rotary evaporator distillation at a temperature of 90 °C and pressure of 30 mbar to purify the resin before polymerization.

The other two resins were end-functionalized in a similar manner by adding 0.5 mol of methacrylic anhydride to the glycerol based and to the pentaerythritol based resin.

### 2.3. Curing Procedure

Cured samples of three bio-based resins were obtained by mixing the resin with dibenzoyl peroxide (2 wt %) as the free radical initiator and *N,N*-dimethylaniline (0.5 wt %) as the accelerator. The mixtures were then left to cure in molds at room temperature for 1 h. Later, the samples were post-cured at 130 °C for 8 h in a drying oven.

The samples were then ground to achieve smooth and flat surfaces required for standard testing dimensions.

### 2.4. Characterization

The polycondensation reaction, resins’ functionalization with methacrylic anhydride and the crosslinking reactions were investigated through Fourier transform infrared spectroscopy (FT-IR) characterization. The resins were tested during the first stage and after the second stage of the synthesis. The cured resins were analyzed using FT-IR spectroscopy using a Nicolet™ iS™ 10 spectrometer supplied by Thermo Fisher Scientific.

The samples were dissolved in CDCl_3_ (δ = 7.26 ppm) and analyzed using ^1^H-NMR (proton nuclear magnetic resonance) spectroscopy on a Bruker (New Delhi, India) make Ascend 400 spectrometer.

The cured resins were also analyzed by differential scanning calorimetry (DSC) using a TA Instrument Q2000 supplied by TA Instruments (New Castle, DE, USA). Samples of cured resin were placed in sealed aluminum pans and heated from 0 °C to 200 °C at 10 °C /min in a nitrogen atmosphere. The glass transition temperature (T_g_) of the cured samples was determined. Tests were replicated at least three times.

Thermogravimetric analysis (TGA) on the cured resins was done on a TA Instrument Q500 supplied by TA Instruments to investigate the thermal stability of the resins. Samples of about 20 mg were heated from 0 °C to 600 °C at a heating rate of 10 °C/min in a nitrogen purge stream and the percentage weight losses of the cured samples were recorded. At least three measurements were done for each sample.

The tensile tests were conducted on the cured resin samples according to ISO 527. Standard dog bone shaped specimens were tested on a Tinius Olsen’s H10KT machine supplied by Elastocon (Borås, Sweden). The machine was equipped with a 2.5 kN load cell and an extensometer. All the tests were performed at a crosshead speed of 1 mm/min. Ten specimens were tested for each batch and the average is reported.

Microscopic analysis was performed on the bio-based cured resin samples using an SMZ800 optical microscope supplied by Nikon Instruments (Stockholm, Sweden).

Thermo-oxidative ageing was performed on the cured resin samples using a climate chamber (NUVE, TK120). The tests were done at a temperature of 40 °C with 75% relative humidity for 695 h. The samples were then dried at 70 °C for 24 h and the dried samples were characterized by FT-IR and the tensile test. Ten specimens were tested for each batch and the average is reported.

The biodegradation analysis was carried out by a standard soil burial test [23]. The specimens were buried in pots between two layers of soil, after being wrapped by plastic nets. The pots were kept at outdoor ambient conditions for 2 months between March and May 2019 (Borås, Sweden). The samples were visually inspected before microscopic analysis every 15 days. After 2 months, the samples were carefully cleaned with distilled water before being dried at 70 °C for 24 h. The samples were then characterised by microscope and DSC.

## 3. Results and Discussion

The purposes of this study were to synthesize thermoset resins with different chemical architectures and to study how hydrolytic degradation and biodegradation differ among them. Three different resins were synthesized with two, three and four arms.

### 3.1. FT-IR Spectroscopic Analysis

**Figure 4:**
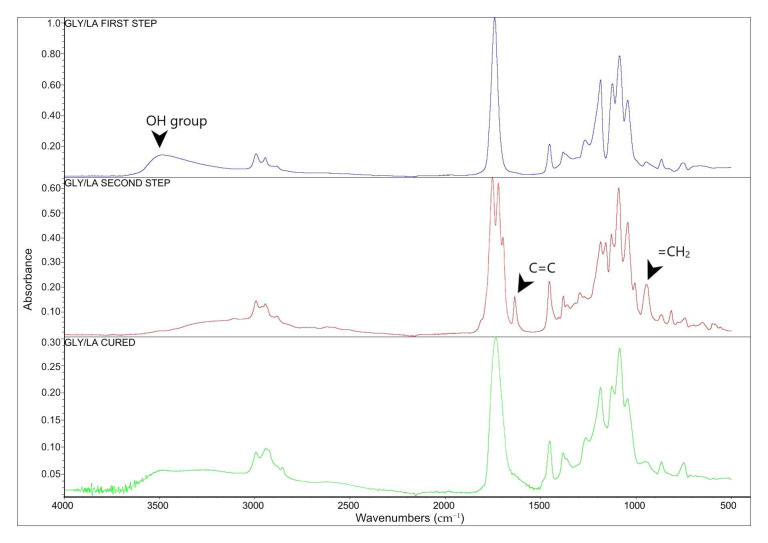
FT-IR spectra of GLY/LA The spectra show the polymer after the first step (top), after the second stage (middle) and the cured resin (bottom).

The Fourier transform infrared (FT-IR) spectroscopy was used to validate the polycondensation reaction and the resin end-functionalization. Figure 4, FT-IR spectra, shows the prepolymer (Gly/LA) after the polycondensation, after the end-functionalization and the cured resin. The other two resins showed very similar spectra and are not included. The spectrum of the prepolymer from the first step is dominated by the large peak at 1722 cm^−1^ which is assigned to the carbonyl group of the formed ester bonds. A broad peak at 3500 cm^−1^ can be seen, which came from the hydroxyl end-terminated polymer. Peaks assigned to SP3-hydridised carbons (–C–H stretch) can be seen slightly below 3000 cm^−1^. When the resin is end-functionalized, carbon–carbon double bonds are introduced which can be seen at about 1640 cm^−1^ (C=C stretch) and CH_2_ at 816 cm^−1^ (C=C, bending) [23,24]. Besides that, hydroxyl groups (–OH) were seen in the first stage, but disappeared, giving further evidence for the end-functionalization.

When the resin from the second step was cured, the peaks at 1640 and 816 cm^−1^ disappeared, showing that the double bonds had reacted in the free radical reaction.

### 3.2. Nuclear Magnetic Resonance Spectroscopic Analysis (NMR)

**Figure 5:**
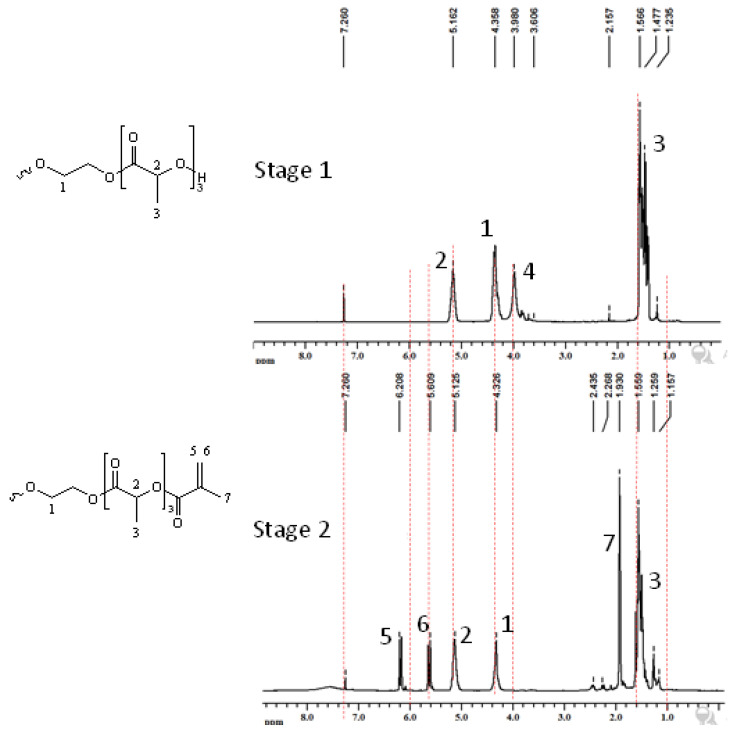
^1^H-NMR of products of reaction 1: **stage 1**—product of ethylene glycol and lactic acid; **stage 2**—product of methacrylic anhydride and stage 1 product.

The products of reaction between ethylene glycol and lactic acid, i.e., stage 1 followed by reaction with methacrylic anhydride (stage 2), were analyzed using ^1^H-NMR, and the peaks assigned to respective protons present in the products are shown in Figure 5. The protons in the –CH_3_ group can be seen at 1.53 ppm, the protons of butanediol can be seen at 4.4 ppm and–C–H can be seen at 5.5 ppm [10]. The peak at 3.9 ppm appeared due to the –OH group of polymerized lactic acid, which upon reaction with MA disappeared in both the first and second stages of reaction. New peaks at 5.6 and 6.2 ppm due to =CH_2_ protons and at 1.9 ppm due to the –CH_3_ group of MA again confirmed the reaction of MA with LA [23].

**Figure 6:**
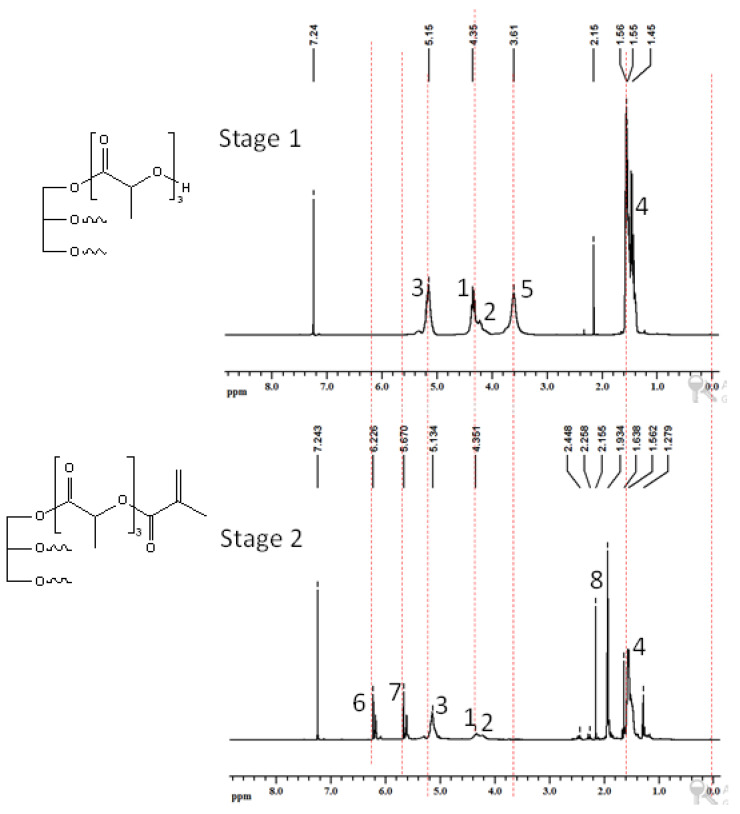
^1^H-NMR of products of reaction 2: **stage 1**—product of glycerol and lactic acid; **stage 2**—product of methacrylic anhydride and stage 1 product.

**Figure 7:**
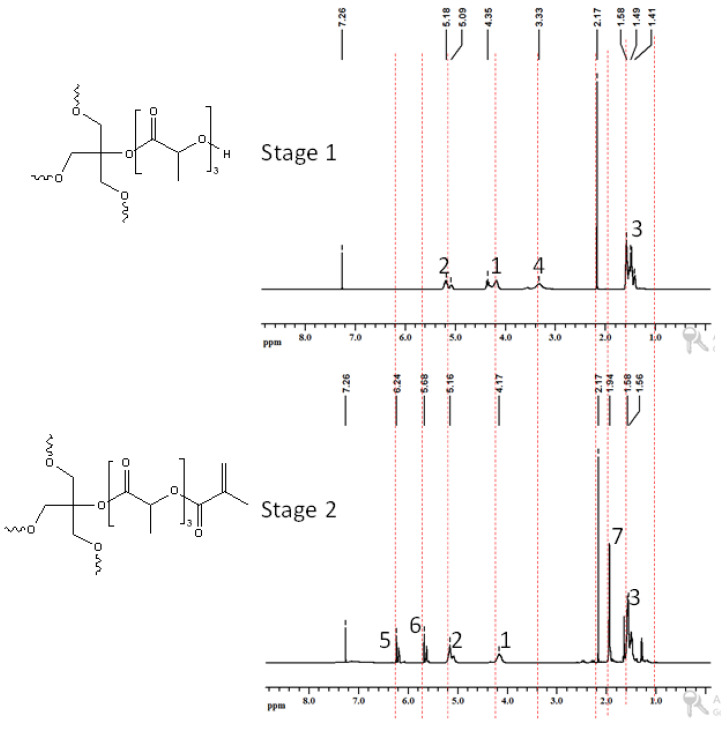
^1^H-NMR of products of reaction 3: **stage 1**—product of pentaerythritol and lactic acid; **stage 2**—product of methacrylic anhydride and stage 1 product.

Similar NMR spectra were obtained when glycerol was used as a core molecule—Figure 6. The protons of glycerol can be seen at about 4.3 ppm [10]. Appearance of peak due to –OH group of polymerized lactic acid in stage 1 of the reactions confirmed the product formation in stage 1 of both reactions. Disappearance of the –OH peak upon reaction with MA confirmed the formation of product of stage 2 in both reactions. This was supported by the appearance of additional peaks at 5.6 and 6.2 ppm due to =CH_2_ protons and at 1.9 ppm due to the –CH3 group of MA. Finally, the resin with pentaerythritol is shown in Figure 7. The protons of pentaerythritol can be seen at about 4.2 ppm. Again, the peak from the -OH disappeared upon reaction with MA, and the introduction of the carbon–carbon double bonds appeared as peaks at about 5.7 ppm and 6.2 ppm.

### 3.3. DSC Analysis

**Figure 8:**
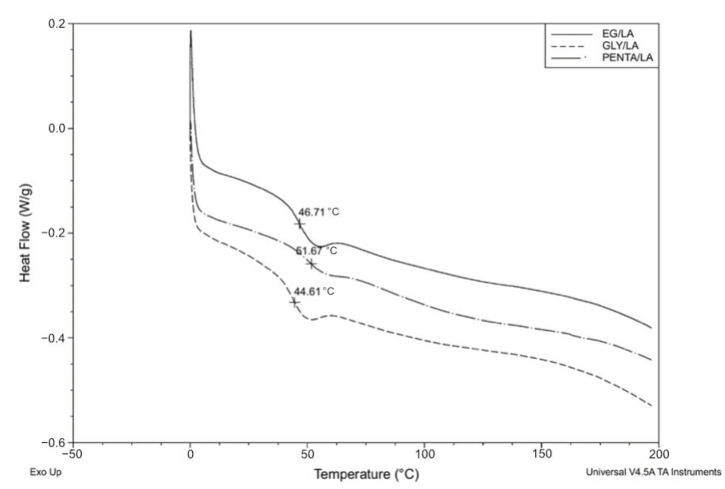
DSC thermograms for the cured resins.

The glass transition temperatures (T_g_) of the resins varied between 44 and 52 °C; see Table 1. PENTA/LA resin had the highest T_g_ when compared to EG/LA and GLY/LA resins. Similar work on bio-based resins exhibited T_g_ in this range [25,26]. Crosslinking of the uncured resin was observed from the exotherms. The crosslinking reaction efficiency of the cured resins was investigated by detecting any residual exothermal heat present in the samples. The cured resins showed no exothermic peak, signifying the complete crosslinking of the resin samples. Figure 8 shows the DSC thermograms of the cured resins.

### 3.4. TGA Analysis

**Figure 9:**
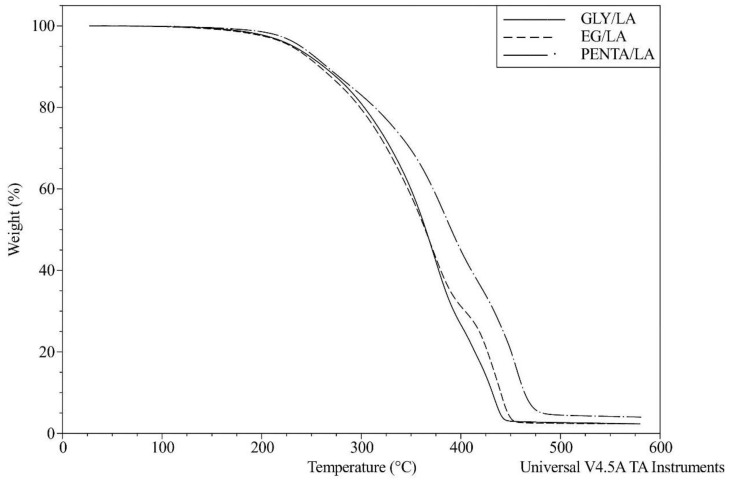
Weight reduction curves from TGA for the cured resins.

**Figure 10:**
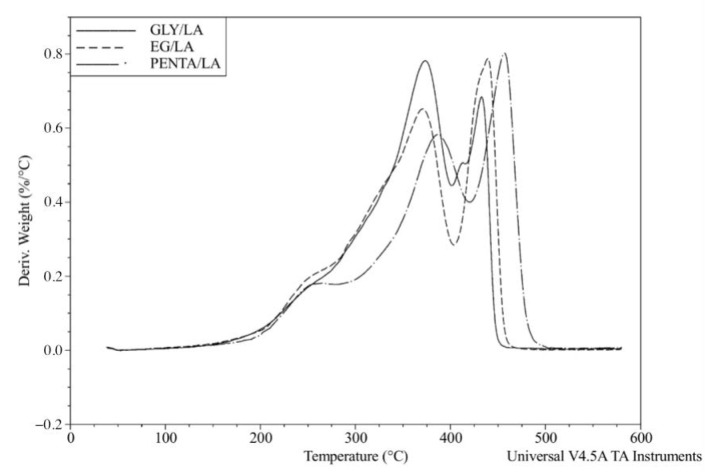
Derivative weight change curves from TGA for the cured resins.

The TGA analysis was performed to investigate the thermal stability and degradation behaviors of the resins by recording the percentage weight losses of the cured samples. All the resins showed high thermal stability, losing 10% of their weight at around 260 °C and 50 wt % at around 370 °C. Table 1 presents the TGA results and it shows that the PENTA/LA resin had the highest thermal stability. The results were comparable to similar bio-based thermoset resins [1]. The degradation of thermoset resins occurred after decomposition of the crosslinked polymer network and the random scission of the linear polymer chains. Increasing the chain length can improve the thermal stability of these resins, as shown in our previous work [1]. Figure 9 and Figure 10 show the weight reduction curves and derivative weight change curves of the cured samples.

### 3.5. Tensile Test

Mechanical properties were characterized by conducting a tensile test on the cured samples. Tensile strength, E-modulus and percentage of elongation are presented in Table 2. There was no significant difference in the tensile strength of the bio-based resins. However, the tensile modulus of the PENTA/LA was highest among three resins. It was also noted that the percentage elongation was less than 0.6% for all the resins. This was an expected outcome, as the unreinforced cured thermoset polyester resins are usually brittle. The linear structure of EG/LA and the star-shaped GLY/LA and PENTA/LA influenced the polymer chain cross-linking, which successively affected the mechanical properties.

### 3.6. Ageing

**Figure 11:**
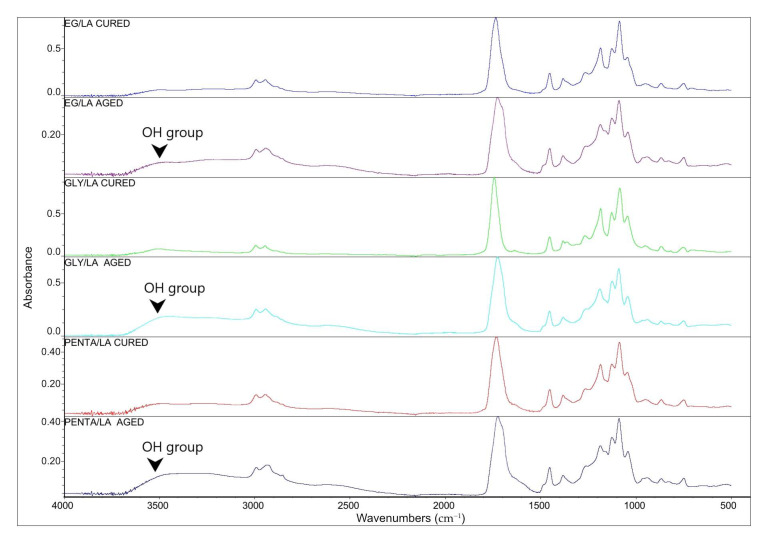
FT-IR spectra of EG/LA, GLY/LA and PE/LA cured resin samples and the same samples after the ageing process.

Thermoset polymers were susceptible to decomposition at an elevated temperature in oxidative environments. Thermo-oxidative ageing was performed by subjecting the samples to a temperature of 40 °C and a relative humidity of 75% for 695 h using a climate chamber. By the end of the ageing process, all the samples underwent physical and chemical ageing and the specimens were malleable, indicating the change in ductility of the samples. This is due to the water plasticizing effect on the polymer molecules affected by the hydrolytic scission of the polymer chains. Such a tendency was noticed in similar work [15,27]. The chemical structural changes resulted from high temperature, oxygen and high humidity combined. The samples then were dried at 70 °C for 24 h and later characterized using FT-IR analysis, DSC, TGA and the tensile test. Figure 11 shows the spectra of the cured resins before and after the ageing process. The appearance of hydroxyl groups after the ageing process was noticed in all three samples as a result of hydrolysis during the ageing. This indicates that the resins were hydrolyzed after the ageing, resulting in more hydroxyl groups being present.

The degradation mechanism of polymers subjected to thermo-oxidative ageing involved surface degradation and micro-cracks perpendicular to the surface, and thus the tensile properties were expected to deteriorate. The degradation occurred on the surface initially and proceeded inwards caused by a diffusion mechanism. Due to this, the micro-cracks appeared perpendicularly to the surface. These micro-cracks increased the surface area and diffusion paths, and therefore enhanced the rate of degradation over time. However, it is important to keep in mind that these results came from the surfaces of the specimens, and the degree of oxidation and subsequent degradation influenced by oxygen diffusion into the specimens. Thus, the results from the standard test dimensions having low surface area to volume ratios are inflated in comparison to the real life structural objects.

The tensile strengths and moduli of all the cured resins decreased significantly after ageing; see Table 3. This was expected due to the degradation mechanism. EG/LA resin was the least affected among the resins. The change in the ductility due to ageing was evident with the increase in elongation of GLY/LA by double and that of PENTA/LA by three times upon ageing. This was most likely caused by water molecules plasticizing the resins, resulting in more ductile materials. The decrease in the modulus and the tensile strength could be influenced by the hydrolytic scission of the polymer chains.

### 3.7. Biodegradation

Soil burial tests are important to assess the biodegradability in various environments. Traditional thermosetting resins are usually produced from petroleum resources and these resins generally degrade slowly in nature. Biodegradability depends not only on the polymer’s origin but also on the chemical structure and the environmental conditions. The end products may vary depending upon the degradation pathway. Bio-based thermoset resins are under development and the biodegradation of these resins may vary quite substantially, depending on several factors, such as the test conditions, the chemical structure and how tightly crosslinked the resin is. Chen et al. synthesized thermoset resins based on soybean oil and vanillyl alcohol. Soil burial tests were done for three months. It was concluded that the resins had good biodegradability [28]. Chow, Tan and Ahmad prepared a bio-based thermoset resin by crosslinking an epoxidized soybean oil with an organic anhydride as a curing agent. The soil burial showed that the resin was significantly degraded after the test [29,30]. Thermoplastic PLA has also been characterized by soil burial tests [31]. In a recent study, Siakeng et al. carried out soil burial tests where both neat PLA and PLA reinforced with coir and pineapple leaf fibers were buried in natural soil [32]. Neat PLA showed a very low biodegradability while the reinforced PLA degraded more quickly.

**Figure 12:**
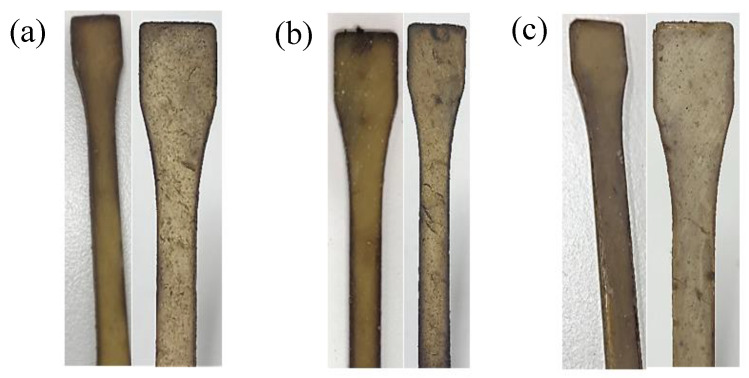
Visual aspects of the resin samples before and after the biodegradation process: (**a**) EG/LA; (**b**) GLY/LA; (**c**) PENTA/LA.

The three resins synthesized in this study were exposed to a soil burial test for two months. The change in the physical appearance on the surface of the cured resins was visible to the naked eyes, Figure 12. The weight reductions of EG/LA and GLY/LA were 7.7% and 8.5% respectively and the weight reduction was primarily due to oxidation and hydrolysis; see Table 4. It is possible that the relatively modest change in weight detected in this study was caused by a relatively high crosslinking density. Thus, microorganisms present in the soil could not penetrate deeply into the polymer in two months. However, there was a thin superficial oxidized layer. This minor change in the cores of the resin samples deterred significant decreases in the glass transition temperatures of these samples; see Table 5.

**Figure 13:**
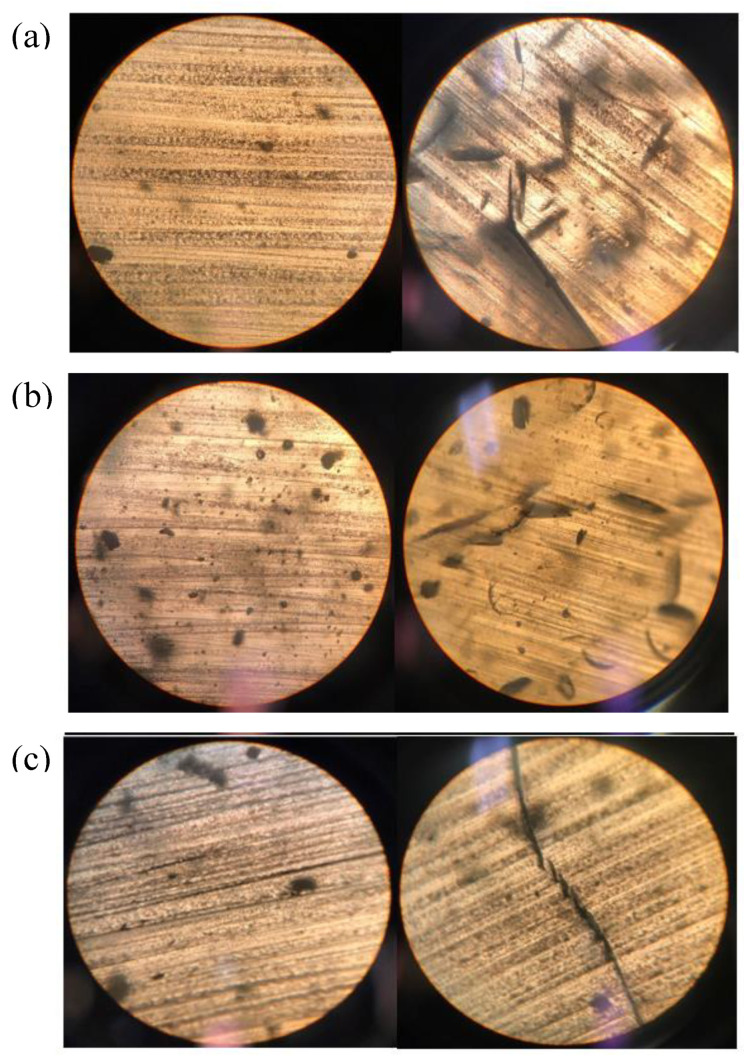
Microscopic images of the resin samples before and after the biodegradation process: (**a**) EG/LA; (**b**) GLY/LA; (**c**) PENTA/LA.

Figure 13 shows the microscopic images of the samples before and after the soil test. The surface morphologies of the resins became considerably rougher as the degradation reactions progressed. The cracks on the surface became visible on the surfaces of the biodegraded resin samples. EG/LA resin sample was the most affected among three different resin samples.

## 4. Conclusions

Three different bio-based thermoset resins from lactic acid were synthesized and characterized to investigate their mechanical, chemical and thermal properties. The process of resin synthesis involved two steps, namely, direct condensation and end-functionalization. In the polycondensation stage, the lactic acid was directly reacted with ethylene glycol, glycerol and pentaerythritol. The resulting intermediate product was then end-functionalized with methacrylic anhydride to obtain three different bio-based unsaturated polyester resins. It was observed that the three resins had, despite having different chemical structures, relatively similar mechanical properties. Ageing and biodegradation tests showed that all three bio-based resins were affected significantly. This was expected, as polyester resins are prone to hydrolysis. A possible application for the resins synthesized would be as matrixes for natural fiber composite applications. As the hydrolytic stability of the resins is limited, the resins could be used for indoor natural fiber composite applications.

## Figures and Tables

**Table 1:** DSC and TGA results of the bio-based thermoset resins.

Resin	DSC	TGA				
	T_g_ (°C)	Degradation Temperature at 10 wt % Loss (°C)	Degradation Temperature at 50 wt % Loss (°C)	Residue (%)	Maximum Degradation (°C)	Second Derivative Peak (°C)
**EG/LA**	46.99 ± 2.22	259.38 ± 4.23	364.81 ± 0.79	1.67 ± 1.42	370.25 ± 0.69	440.09 ± 0.44
**GLY/LA**	44.39 ± 2.54	264.48 ± 6.90	365.65 ± 1.50	2.19 ± 0.25	371.99 ± 0.39	433.79 ± 1.45
**PENTA/LA**	51.81 ± 2.68	262.44 ± 5.70	390.05 ± 2.07	3.80 ± 0.18	382.34 ± 1.83	457.02 ± 0.71

± stands for the standard deviation.

**Table 2:** Tensile properties of the bio-based thermoset resins.

Resin	Tensile Modulus (GPa)	Tensile Strength (MPa)	Maximum Elongation (%)
**EG/LA**	2.32 ± 0.18	12.03 ± 2.64	0.58 ± 0.13
**GLY/LA**	3.76 ± 0.52	11.08 ± 3.27	0.32 ± 0.11
**PENTA/LA**	3.80 ± 0.91	13.15 ± 4.99	0.39 ± 0.22

± stands for the standard deviation.

**Table 3:** Tensile properties of the bio-based resins after ageing.

Resin	Tensile Modulus (GPa)	Tensile Strength (MPa)	Maximum Elongation (%)
**EG/LA**	1.82 ± 0.05	6.78 ± 1.61	0.43 ± 0.11
**GLY/LA**	0.83 ± 0.05	4.59 ± 2.12	0.74 ± 0.43
**PENTA/LA**	0.50 ± 0.12	4.68 ± 0.77	1.48 ± 0.77

± stands for the standard deviation.

**Table 4:** Effects of biodegradation on weights of the cured resins.

Resin	Weight before Biodegradation (g)	Weight after Biodegradation (g)
**EG/LA**	1.18 ± 0.01	1.09 ± 0.02
**GLY/LA**	1.19 ± 0.04	1.09 ± 0.05
**PENTA/LA**	1.18 ± 0.01	1.16 ± 0.02

± stands for the standard deviation.

**Table 5:** Glass transition temperatures of degraded samples.

Resin	Glass Transition Temperature, Tg (°C)
**EG/LA**	43.90 ± 2.09
**GLY/LA**	41.86 ± 3.33
**PENTA/LA**	43.42 ± 2.54

± stands for the standard deviation.
